# Tiny Troublemakers—A Comprehensive Approach to Crusted Scabies

**DOI:** 10.3390/diagnostics15060680

**Published:** 2025-03-10

**Authors:** Antonia Armega-Anghelescu, Raluca-Maria Closca, Daliborca-Cristina Vlad, Florentina-Camelia Cioenaru, Marina Rakitovan, Patricia Cristodor, Caius-Silviu Solovan, Marco-Cristian Marian, Maria-Bianca Ilas-Tat, Flavia Zară

**Affiliations:** 1 Department of Pathology, Emergency City Hospital, 300254 Timisoara, Romania; antonia.armega@umft.ro (A.A.-A.); camelia.cioenaru@yahoo.com (F.-C.C.); drtatbianca0@gmail.com (M.-B.I.-T.); flavia.zara@umft.ro (F.Z.); 2 Department of Microscopic Morphology, “Victor Babes” University of Medicine and Pharmacy, 300041 Timisoara, Romania; marina.rakitovan@umft.ro; 3 Department of Pharmacology and Biochemistry-Pharmacology, “Victor Babes” University of Medicine and Pharmacy, 300041 Timisoara, Romania; vlad.daliborca@umft.ro; 4 Oro-Maxillo-Facial Surgery Clinic, Emergency City Hospital, 300062 Timisoara, Romania; 5 Department of Dermatology, Emergency City Hospital, 300254 Timisoara, Romania; patricia.cristodor@gmail.com (P.C.); caius.solovan@gmail.com (C.-S.S.); 6 Department X, 2nd Surgical Clinic, Researching Future Chirurgie 2, “Victor Babes” University of Medicine and Pharmacy, 300041 Timisoara, Romania; marian.marco@umft.ro

**Keywords:** scabies, crusted scabies, hypereosinophilia, anaphylaxis

## Abstract

**Background and Clinical Significance:** The current paper presents a retrospective case of a 79-year-old female patient admitted to the Dermatology Clinic of Emergency City Hospital in Timisoara, Romania, in January 2022, reporting intense pruritus and burning sensation of the skin exacerbated at night. **Case Presentation:** The previously mentioned symptoms appeared approximately six months prior, with gradual and continuous progression. Clinical examination revealed widespread hyperkeratosis on diffuse erythematous background across the entire body, accompanied by crusted lesions predominantly on the arms and legs. Laboratory values showed elevated absolute eosinophil count as well as a positive culture swab to *Staphylococcus aureus*. Two incisional skin biopsies were performed. Microscopic examination in Hematoxylin–Eosin staining revealed thickened stratum corneum with numerous oval-shaped mites with exoskeleton and striations and moderate perivascular lympho-eosinophilic infiltrate in the superficial dermis, leading to a positive diagnosis of crusted scabies. Following etiological treatment, the patient’s evolution was undulating and on the 10th day of hospitalization presented marked dyspnea, followed by cardiorespiratory arrest, leading to the patient’s death. **Conclusions:** The patient’s outcome could be explained by a Th2-mediated allergic response to *Sarcoptes scabiei* allergens, in addition to the presence of *Staphylococcus aureus* on the damaged skin, as dysbiosis can further support an uncontrolled Th2 reaction, leading to anaphylaxis.

## 1. Introduction and Clinical Significance

Scabies is a parasitic dermatosis caused by the ectoparasitic mite *Sarcoptes scabiei* var. hominis, affecting 200 million people worldwide [1]. Based on clinical presentation, it is categorized into three primary subtypes: classic scabies, crusted scabies, and bullous scabies [2]. Classic scabies, the most prevalent form, is characterized by a moderate mite burden, intense pruritus, typically nocturnal, alongside erythematous papules, burrows, and in some cases nodules, ranging in color from skin-toned to red-brown or violaceous, developed as a result of exacerbated hypersensitivity and frequent scratching [2,3,4,5]. The most severe type is bullous scabies, characterized by bullae and crusts that closely resemble those found in bullous pemphigoid disease [3].

Multiple defense mechanisms work in order to eliminate scabies mites through both mechanical and immunological processes such as proper hygiene and scratching, can lead to decreasing the mite burden [5]. An immune response to mite antigens and products typically develops three to six weeks post-infestation [5]. Compromised or absent defense mechanisms can induce crusted scabies—also referred to as Norwegian scabies, a rare but severe variant—more commonly observed in patients receiving topical or systemic corticosteroids, immunotherapy or biological agents, as well as in elderly, disabled, and debilitated patients [4,5,6]. Incidence of crusted scabies is reported to be around 0.5% [2].

This form is marked by a significantly higher mite presence in the stratum corneum, potentially impacting various areas of the skin’s surface, leading to intense skin infestation due to dysregulated keratin production [5,6,7,8,9]. It is characterized by extensive local or disseminated hyperkeratosis on an erythematous background, accompanied by crusting predominantly on the hands, feet, trunk, neck, and elbows [5,6,7,8,9]. Nail involvement typically includes thickening, discoloration, and dystrophy [5]. The proliferation of thousands, or even millions, of mites contribute to the formation of prominent scales, making crusted scabies highly contagious. Scabies can be transmitted via direct or indirect contact with contaminated materials, such as bedding or clothing [5,6,7,8,9].

Unlike classic scabies, crusted scabies is associated with a higher mortality rate, culminating up to 50% in the last five years, due to secondary bacterial infections or sepsis [6,10]. Clinical presentation can be complicated by non-specific secondary lesions, including excoriated papules, as well as eczematous or lichenified plaques, with patients typically reporting generalized itching that is exacerbated at night [7]. Despite the common belief that pruritus is absent in crusted scabies, nearly half of the patients diagnosed with this condition report varying degrees of pruritus, which typically subsides over time [11,12]. Generalized lymphadenopathy, eosinophilia, and elevated Immunoglobulin E (IgE) titer can be found as well [1,5,7].

Atypical or extreme clinical presentations may lead to misdiagnosis, as scabies can mimic other dermatological conditions; in particular, crusted scabies may resemble other causes of erythroderma due to its exuberant scaling and crust formation [1,13,14,15]. Secondary infections caused by *Streptococcus pyogenes* and *Staphylococcus aureus* are common complications that can lead to toxin-mediated diseases such as scarlet fever, toxic shock syndrome, and post-streptococcal glomerulonephritis [1,13,14,15,16].

This article presents the case of a 79-year-old female patient with crusted scabies. The initial clinical diagnosis was of cutaneous lymphoma, with similar clinical appearance, manifesting erythematous papules, nodules, patches, plaques, and occasional erythroderma [17,18]. The aim of this study is to bring awareness to a scabies variant which potentially led to anaphylaxis and a poor outcome.

## 2. Case Presentation

The current paper is a retrospective presentation of a 79-year-old female patient admitted to the Dermatology Clinic of Emergency City Hospital Timisoara, Romania, in January 2022, complaining of intense pruritus and burning sensation of the skin.

**Figure 1:**
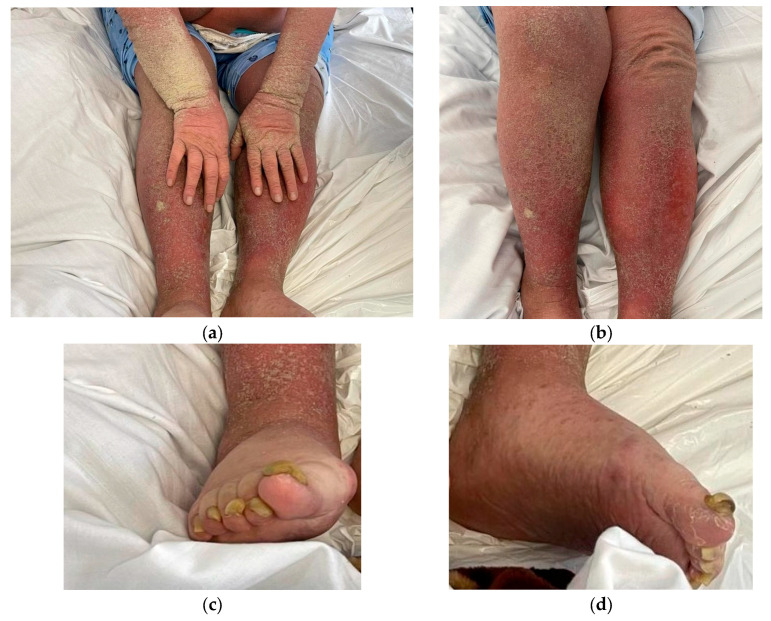
Clinical presentation: (**a**) Hyperkeratosis with dry crusts on arms, hands and legs; (**b**) erythematous rash causing intense pruritus; (**c**,**d**) nail involvement (onychogryphosis).

Based on the initial examination, the patient was conscious but disoriented. Dermatological status showed diffuse erythematous lesions and widespread hyperkeratosis, accompanied by crusting, predominantly on the arms and legs, causing pruritus and burning sensation of the skin (Figure 1). Anamnestic, the patient established the occurrence of symptoms approximately 6 months prior, with a slow continuous progression. Additionally, nail involvement included onychogryphosis.

Clinical examination also revealed peripheral edema affecting the feet and ankles, as well as shortness of breath and loss of spatial awareness. Further consultations suggested hypertension, congestive heart failure, and mixed dementia. Based on clinical aspects, an initial presumptive diagnosis of erythroderma was established. A possible alternative to the initial diagnosis of cutaneous lymphoma was also considered. The patient was directed for further testing.

Laboratory testing on admission demonstrated a high-persistent peripheral blood eosinophil count of 19.3% with absolute eosinophil count of 1650 cells/µL and elevated C-reactive protein level of 115.6 mg/L. Additional examinations were performed for this patient, including RT-PCR (Reverse transcription–polymerase chain reaction) assay for SARS-CoV-2 with a negative result and a culture of wound swab with positive result for Methicillin-susceptible *Staphylococcus aureus*. A pharyngeal swab was also performed, with negative results for *Streptococcus pyogenes* and *Staphylococcus aureus.* Antistreptolysin O (ASO) titer was also within normal range.

Two further incisional skin biopsies were performed. The harvested tissue, fixed in 10% (*v*/*w*) neutral buffered formalin, was sent to the Department of Pathology of Timisoara Emergency City Hospital for a histopathological examination. The gross examination of the two harvested skin specimens of 1.2/0.5/0.2 mm and 1.4/0.5/0.4 mm, respectively, revealed two tissue fragments with epidermis covered with white hyperkeratotic plaques with no other macroscopic changes. The two fragments were processed as follows: the first biopsy specimen was sectioned into three consecutive fragments, while the second one was sectioned into two complementary fragments; all were further processed for histopathological examination.

**Figure 2:**
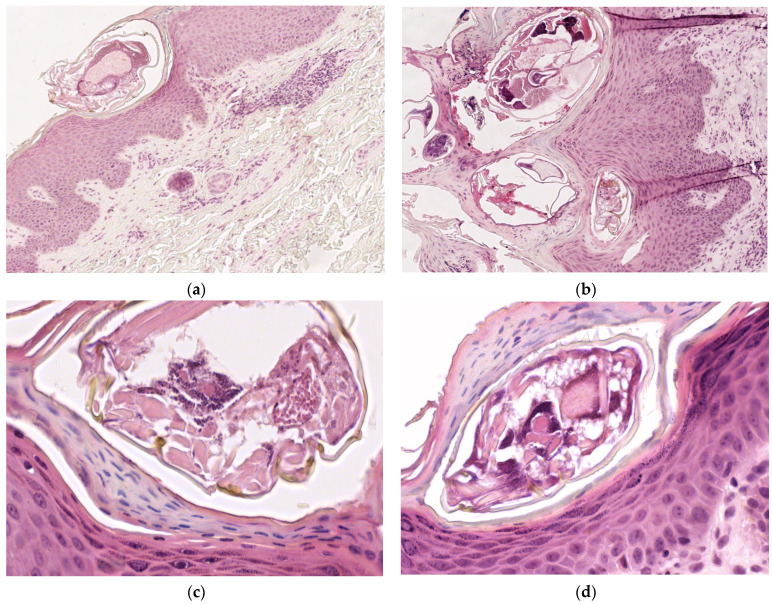
Microscopic examination on Hematoxylin–Eosin-stained slides: (**a**) *Sarcoptes scabiei* mite in stratum corneum, ob. 10×; (**b**) intracorneal mites, eggshells, and fecal deposits (scybala), ob. 10×; (**c**,**d**): oval shaped mites of 0.2 mm length and exoskeleton with striations, ob. 40×.

Four-micrometer-thick serial sections were prepared for the diagnosis from paraffin blocks, using morphological Hematoxylin–Eosin (HE) staining. The microscopic examination of Hematoxylin–Eosin-stained slides revealed epidermal acanthosis and focal hyperkeratosis, with the identification of numerous oval shaped mites of 0.2 mm length and exoskeleton with striations. Additionally, moderate perivascular lympho-eosinophilic infiltrate was found in the superficial dermis (Figure 2).

Based on patient history, dermatological examination and histopathological aspects on HE-stained slides, the final diagnosis was crusted scabies. The patient was admitted and treated as per guidelines with oral ivermectin 200 µg/kg on days 1, 2, and 8, as well as 10% precipitated sulfur ointment daily. Initial laboratory values showed high peripheral blood eosinophil count of 19.3% and absolute eosinophil count of 1650 cells/µL. On day 6, the peripheral blood eosinophil count was 17.3%, a slight decrease compared to initial values. There was also an increase in the patient’s total white blood cell count, from an initial value of 8560 cells/µL to 10,340 cells/µL.

Considering the positive result for Methicillin-susceptible *Staphylococcus aureus,* an antibiogram was performed in order to guide a proper antimicrobial therapy. The patient was subsequently treated with oxacillin 500 mg every 6 h. Following etiological treatment, the patient’s evolution was undulating and, on day 10, culminated with marked dyspnea followed by cardiorespiratory arrest. As the patient’s symptoms indicated an acute anaphylactic reaction, adrenaline (epinephrine) 0.5 mg was promptly administered intramuscularly and repeated after 5 min, as well as the administration of hydrocortisone hemisuccinate 200 mg intravenously; however, the patient was ultimately unresponsive.

## 3. Discussion

The first documented case of crusted scabies was published in 1848 by Danielsen and Boeck. Since the condition was initially identified in a patient with leprosy in Norway, it was originally called “Norwegian scabies” [19]. Subsequent reports associate crusted scabies with renal insufficiency [20], chronic corticosteroid therapy, Down’s syndrome, malnutrition, and patients with cognitive deficiency, as they are less able to properly interpret or respond to the itch caused by the mites [21]. Norwegian scabies is also linked to T-cell dysregulation (organ transplantation, HIV infection, lymphoma) [22].

Initial physical examination of the patient’s skin showed diffuse erythematous lesions and widespread hyperkeratosis, accompanied by crusting across nearly the entire body surface, predominantly on the arms and legs and onychogryphosis. This aligns with the theory that the initial lesions in Norwegian scabies presented as ill-defined, erythematous patches that progressed into diffuse hyperkeratotic plaques [23]. Nail abnormalities, such as hyperkeratotic or dystrophic nails, along with thick, psoriasis-like scales accumulating beneath the nails, are hallmark features of this condition. Mites often survive within the subungual material, thus increasing the risk of re-infestation [24]. These symptoms may be accompanied by generalized redness and scaling, often resembling erythroderma [12], which corresponded with the patient’s initial presumptive diagnosis. Standard dermoscopy may be utilized for clinical diagnosis of *Sarcoptes scabiei* infection [25]. However, if the scabies mites cannot be detected through standard dermoscopy, ultraviolet (UV)-dermoscopy may be performed, providing clearer and improved images compared to polarized dermoscopy [26]. Keratin serves as a primary source of fluorescence under UV-dermoscopy [25]. The female mite secretes keratolytic enzymes to facilitate burrow formation [27]. Further studies in electron microscopy have also revealed keratin flakes partially covering the mite’s dorsal surface; additionally, the distal portion of the burrow consists of keratinic collarettes [27]. Keratin emits a distinct yellow fluorescence when exposed to Wood’s lamp, highlighting the contents of the tunnels produced by the mites as a bright reflection [25].

As a result of a positive scabies diagnosis, the patient was isolated as they could be a source of an epidemic outbreak. Permethrin (as a 5% concentration cream or lotion) has been considered the gold standard treatment for scabies, yet recent studies have shown a high resistance of mites to this medication. However, it is important to note that some studies suggest that this resistance may not be accurate and that the ineffectiveness may be due to application errors [4,12]. The crusted lesions may also hinder the efficacy of topical agents; therefore, keratolytic agents may improve drug penetration for topical scabicides. Topical Lindane should be avoided, as increased percutaneous absorption may potentially cause fatal neurotoxicity [22].

As for the treatment of crusted scabies, one guideline recommends the use of a topical scabicide applied daily for seven days, followed by applications two times a week until resolution; oral ivermectin at a dose of 200 µg/kg on days 1, 2, and 8 may be considered, with additional doses on days 9 and 15, or alternatively on days 9, 15, 22, and 29 for severe or persistent infestations. This recommendation is based on expert committee reports and clinical experience of respected authorities [28].

Ivermectin can be highly effective in crusted scabies through a mechanism GABA (gamma-aminobutyric acid) receptor blockade [29]. One older study from 1997 by Barkwell et al. [30] involving patients from a long-term care facility in Ontario, Canada revealed increased statistical correlation between the use of ivermectin and an elevated risk of death in elderly patients; however, to the best our knowledge, no further studies were able to demonstrate this finding.

Following the treatment, the patient’s outcome was poor. A possible cause of death may be linked to our patient’s positive swab culture of methicillin-susceptible *Staphylococcus aureus*, as it may lead to a systemic bacterial infection.

According to Swe and Fisher [31], there may be evidence of life-threatening complications caused by *Staphylococcus aureus* associated with scabies infestation, due to a facile penetration of bacteria through the damaged skin. Their studies suggest that a protein referred to as SMSB4 (mite serine protease inhibitor molecule), produced by scabies mites as a defense mechanism against the host, is affecting all three of the complement pathways. SMSB4 inhibits the bactericidal activity against various strains of *Staphylococcus aureus*, as it is released into the damaged skin and interferes with the complement-dependent cytotoxic activity of neutrophils, leading to a decreased uptake of *Staphylococcus aureus* by neutrophils and contributing to a possible onset and dissemination of a *Staphylococcus aureus* infection.

In France, Jouret et al. [32] presented a case report of crusted scabies complicated by *Staphylococcus aureus* bacteremia in a 52-year-old female patient leading to multi-organ failure and death. A systematic review by Lee et al. [33] attributed the deaths of scabies patients to their underlying chronic background, while also indicating the possibility of linking their demise to secondary bacterial infections.

An Australian rural hospital demonstrated a decrease in mortality rate from 4.3% to 1.6% following the implementation of an empirical antibiotic protocol for secondary infections alongside ivermectin treatment [21]. Their finding supports the hypothesis that secondary sepsis plays a contributory role in mortality.

Wang et al. [34] discuss their findings regarding the possible causes of acute glomerulonephritis in patients with scabies. According to one of their perspectives, secondary bacterial infections may arise from skin lesions caused by scratching, which subsequently lead to the development of acute glomerulonephritis following a latent period of streptococcal infection. Their study suggests that adults may be affected by glomerulonephritis related to bacterial infections, more likely due to non-streptococcal infections, particularly in staphylococcal infections originating from diverse sites, such as the skin, the upper respiratory tract, the oral cavity, or the urinary tract [34]. Their results list staphylococcal pulmonary infection as the possible cause of acute glomerulonephritis originating from skin lesions caused by scabies mites. As an alternative perspective, they also suggest the possibility of an immune complex-mediated nephritis, due to immune complexes formed by the scabies mites or their products binding with immunoglobulins. Wang et al. [34] were able to demonstrate the deposition of immunoglobulins, complement, and fibrin in the glomeruli, concluding that both bacterial infection and scabies mites may contribute to the development of acute nephritis.

According to Mika et al. [35], scabies mites produce multiple proteins that can affect the complement pathways, most notably two scabies mite serpins (SMSs) and two scabies mite inactive serine protease paralogues (SMIPP-Ss). Both of them serve as potent complement inhibitors, each interacting with a variety of complement factors at multiple stages across the three complement pathways. Their combined activity promotes the survival of bacterial pathogens within the microenvironment of the epidermis, include *Streptococcus pyogenes*. These interactions demonstrated by Mika et al. [35] underscore the potential value of a coordinated intervention against scabies to control secondary bacterial skin infections, suggesting that these complement-inhibitory proteins may serve as novel targets for protective intervention at the onset of both scabies and pyoderma.

Blas et al. [36] suggested the consideration of human T-cell leukemia virus type I (HTLV-I) infection in patients with Norwegian scabies, particularly in endemic regions, when no other evident risk factors were present, revealing that this rarely suspected retroviral infection may also be linked to recurrent crusted scabies episodes or persistent infestation, potentially leading to fatal outcomes due to bacterial infection. Blas et al. [36] reported the fatal outcome for 3 of 23 HTLV-I-positive patients within a few days following the diagnosis, indicating Norwegian scabies as a risk factor, although the actual cause of death was not determined. Findings by del Giudice et al. [37] suggested that the presence of Norwegian scabies in patients seropositive for HTLV-I may indicate significant immunosuppression associated with adult T-cell leukemia/lymphoma, and they proposed careful monitoring of such patients, as Norwegian scabies may precede its onset.

In our case, we initially considered the patient’s clinical evolution and subsequent poor outcome to be a result of a secondary bacterial infection. However, laboratory values did not fully corroborate this theory and our patient still received antimicrobial therapy.

Another plausible cause of death in our patient may be associated with an allergic immune response due to the high mite burden released into the bloodstream following the treatment. According to an older research paper by Prakken et al. [38], histological findings in crusted scabies, particularly vascular changes, demonstrate the allergic nature of the pathological reactions associated with this condition.

Upon the initial exposure to an antigen, dendritic cells (known as antigent-presenting cells of epithelial tissue) process and present the antigen to a CD4+ T-lymphocyte, and it is during this process that the naïve T lymphocyte can differentiate into either a T-helper 2 (Th2) or a T-helper 1 (Th1) lymphocyte [39].

Newer evidence presented by multiple authors [40,41] suggest that Norwegian scabies primarily develops due to a Th1/Th2 imbalance, characterized predominantly by a skin cytotoxic Th2 response, alongside elevated blood antibody levels and uncontrolled parasite proliferation. Walton et al. [40] demonstrated the predomination of infiltrating CD8+ T lymphocytes in the dermis, along with the absence of B lymphocytes in skin biopsies from patients diagnosed with crusted scabies. Their findings suggest that skin-homing CD8+ T lymphocytes could be linked to the inflammatory response, although the role of cytotoxic T cells in Norwegian scabies has not been elucidated. Tissue damage mechanisms could involve direct cytotoxicity against keratinocytes leading to cytokine release, which may further amplify inflammation by targeting resident cells of the skin [40,41]. T lymphocites are essential in the activation and regulation of immune responses, as they recognize antigens and induce the production of cytokines; additionally, keratinocytes produce pro-inflammatory cytokines such as IL-1, IL-6, IL-8, and TNF-α, as well as immunomodulatory cytokines such as IL-10 and IL-12, the latter having been thought to contribute to systemic effects [41].

When the antigen is an allergen, the lymphocyte differentiates into a Th2 cell, which secretes IL-4, stimulating B lymphocytes to produce IgE. In addition to its role in regulating IgE synthesis, IL-4 also promotes the production of IL-13 by mast cells [39]. Eosinophils, basophils, and mast cells are known to play a key role in the initiation and regulation of Th2 responses; these cells may be promptly recruited to infection sites and draining lymph nodes in order to produce IL-4 and/or IL-13 [41,42,43,44]. Skin biopsy sections from Norwegian scabies lesions revealed significant infiltration of eosinophils and lymphocytes in the dermis, accompanied by blood eosinophilia and increased IgE production [41,44].

*Sarcoptes scabiei* mites produce a number of allergens (paramyosin, apolipoprotein, glutathione S-transferase, serine protease, and cysteine protease) [42]. It has been shown that patients with crusted scabies have elevated serum IgE levels against recombinant *Sarcoptes scabiei* cysteine and serine proteases. Combined elevated levels of scabies-specific IgE and IgG4 in the plasma of individuals with crusted scabies, along with the documented clinical eosinophilia in these patients, collectively suggest an increased production of the cytokines IL-4, IL-5, and IL-13 in combination with a non-protective Th2 response [44]. Traditionally associated with responses to extracellular bacteria and parasites, recent studies have emphasized the role of the Th2-mediated immune response in the neutralization of venoms and toxins, as well as in tissue repair following injury; however, Th2-mediated immunity can also result in uncontrolled or maladaptive inflammatory reactions, such as the production of IgE antibodies to allergens [41,44]. A critical factor in Th2-mediated allergic responses is the disruption of the epithelial barrier, as dysbiosis at these sites further supports an uncontrolled Th2 reaction, promoting allergy development [41,42,43,44]. During subsequent allergen exposure, crosslinking of IgE triggers the release of mediators like histamine, leading to immediate vasoactive effects, including potential anaphylaxis [42,43,44,45].

To the best of our knowledge, there is no other article in the current existing literature that presents the hypothesis of an inceased allergic immune reaction to crusted scabies leading to anaphylaxis.

Anaphylaxis is primarily thought to result from the activation of basophils and mast cells through a mechanism that typically involves the crosslinking of immunoglobulin (Ig) E and the aggregation of high-affinity IgE receptors called FcεRI [46]. Mast cells possess the high-affinity IgE receptor (FcεRI) on their membranes and once IgE binds to this receptor, it becomes primed to target the allergen to which it is sensitized; at this stage (the sensitization phase), no clinical symptoms are observed [39]. IgE binds predominantly to the FcεRI receptor on basophils and mast cells but also, to a lesser extent, on other types of cells such as neutrophils, eosinophils, dendritic cells, monocytes, and platelets [46,47]. When two or more IgE molecules, bound to their receptors, recognize the same allergen, they initiate receptor cross-linking—a process that triggers a cascade of biochemical signaling events, inducing the activation of mast cells and basophils, with the rapid release of preformed histamine and protease mediators [39,46,47]. The acute inflammatory response is further favored by the de novo synthesis of various inflammatory mediators, including leukotrienes, prostaglandins, and cytokines. Histamine and cysteinyl leukotrienes in particular play a key role in producing some of the characteristic signs and symptoms of anaphylaxis [47].

Considering recent studies, which have demonstrated that patients with crusted scabies have elevated scabies-specific IgE in their plasma [43], anti-IgE therapy could potentially be of benefit alongside etiological treatment. The effect of omalizumab (a recombinant humanized monoclonal antibody against human IgE) is used to reduce both the amount of free serum IgE and the expression of FcεRI receptors on mast cells and basophils [47,48]. Omalizumab also decreases serum and tissue eosinophilia [47].

The patient’s evolution was unfavorable, presenting on day 10 of hospitalization a marked dyspnea and cardiorespiratory arrest. The poor outcome could be explained by a Th2-mediated acute inflammatory response to *Sarcoptes scabiei* allergens. As discussed previously, an uncontrolled Th2 reaction may have resulted in the activation of mast cells and basophils, leading to mast cell degranulation with the rapid release of preformed mediators and subsequent anaphylaxis.

## 4. Conclusions

Crusted scabies, also called Norwegian scabies, is a rare variant of scabies characterized by a massive mite infestation and is associated with a higher mortality rate, potentially culminating in sepsis, due to secondary bacterial infections.

There is proven histological evidence that explains the clinical and immunological difference between classic scabies and the crusted variant, as well as the pathological immune response in the skin. Patients with crusted scabies show a predomination of infiltrating CD8+ T lymphocytes and eosinophils in the dermis, along with the absence of B lymphocytes, increased IgE production and elevated blood eosinophilic count, collectively suggesting an increased production of the cytokines IL-4, IL-5, and IL-13. The patient’s poor outcome may be attributed to a Th2-mediated immune response to *Sarcoptes scabiei* allergens. Considering the mechanism for an allergic immune response, more frequently studied in recent years, a potential allergic reaction in patients with crusted scabies should be considered. As topical or systemic corticosteroids are among the risk factors for crusted scabies, it may be prudent to avoid steroids as anti-inflammatory treatment. Patients could potentially benefit from treatment with omalizumab alongside etiological treatment; however, further research is needed to confirm this hypothesis.

All things considered, reporting of the uncommon cases of crusted scabies is meaningful for improving the literature, as well as for progressing with our understanding of this rare variant and its overall development and implications.

## Data Availability

The data that support the fundings on this study are available from the corresponding author upon reasonable request.
